# A Computational Modeling Study to Investigate the Use of Epicranial Electrodes to Deliver Interferential Stimulation to Subcortical Regions

**DOI:** 10.3389/fnins.2021.779271

**Published:** 2021-12-16

**Authors:** Ahmad Khatoun, Boateng Asamoah, Myles Mc Laughlin

**Affiliations:** ^1^ExpORL, Department of Neurosciences, KU Leuven, Leuven, Belgium; ^2^The Leuven Brain Institute, KU Leuven, Leuven, Belgium

**Keywords:** epicranial electrode, interferential stimulation, subcortical stimulation, transcranial stimulation, neuromodulation

## Abstract

**Background:** Epicranial cortical stimulation (ECS) is a minimally invasive neuromodulation technique that works by passing electric current between subcutaneous electrodes positioned on the skull. ECS causes a stronger and more focused electric field in the cortex compared to transcranial electric stimulation (TES) where the electrodes are placed on the scalp. However, it is unknown if ECS can target deeper regions where the electric fields become relatively weak and broad. Recently, interferential stimulation (IF) using scalp electrodes has been proposed as a novel technique to target subcortical regions. During IF, two high, but slightly different, frequencies are applied which sum to generate a low frequency field (i.e., 10 Hz) at a target subcortical region. We hypothesized that IF using ECS electrodes would cause stronger and more focused subcortical stimulation than that using TES electrodes.

**Objective:** Use computational modeling to determine if interferential stimulation-epicranial cortical stimulation (IF-ECS) can target subcortical regions. Then, compare the focality and field strength of IF-ECS to that of interferential Stimulation-transcranial electric stimulation (IF-TES) in the same subcortical region.

**Methods:** A human head computational model was developed with 19 TES and 19 ECS disk electrodes positioned on a 10–20 system. After tetrahedral mesh generation the model was imported to COMSOL where the electric field distribution was calculated for each electrode separately. Then in MATLAB, subcortical targets were defined and the optimal configurations were calculated for both the TES and ECS electrodes.

**Results:** Interferential stimulation using ECS electrodes can deliver stronger and more focused electric fields to subcortical regions than IF using TES electrodes.

**Conclusion:** Interferential stimulation combined with ECS is a promising approach for delivering subcortical stimulation without the need for a craniotomy.

## Introduction

The use of electrical brain stimulation to modulate neural activity is a rapidly growing field with many novel methods currently being proposed and evaluated (Lewis et al., 2016; Brittain and Cagnan, 2018; Vöröslakos et al., 2018). One of the more established methods is deep brain stimulation (DBS) which is currently used in the clinic to successfully treat a range of neurological and psychiatric disorders (Lee et al., 2019; Lozano et al., 2019). However, DBS requires a highly invasive neurosurgical procedure to chronically implant electrode arrays in deep brain regions. This increases the invasiveness and brings risks associated with the surgical procedure (Fenoy and Simpson, 2014). Thus, non-invasive electric neuromodulation methods such as transcranial electric stimulation (TES, e.g., transcranial direct current stimulation, transcranial alternating current stimulation, and transcranial random noise stimulation), where electrodes are placed on the scalp, are currently under investigation as non-invasive alternatives (Moreno-Duarte et al., 2014; Bestmann and Walsh, 2017). The use of scalp electrodes means that TES is completely noninvasive. However, this comes at the cost of the electric field strength and focality in the brain because the skin shunts most of the applied current resulting in a weak electric field in the brain. Furthermore, co-stimulation of peripheral nerves in the scalp means that TES becomes painful if amplitudes are increased above 2 mA (Bikson et al., 2016; Antal et al., 2017). In a recent study, we introduced a novel technique, epicranial cortical stimulation (ECS), and demonstrated its potential at overcoming some of the limitation of TES (Khatoun et al., 2019). In Our computational modeling and animal studies ECS induced stronger and more focused electric fields in the cortex than TES (Khatoun et al., 2019). Yet, ECS requires a minimally invasive surgical intervention to place stimulation electrodes under the scalp and directly over the skull. There is currently an ongoing clinical trial using ECS electrode in epilepsy patients (EASEE, 2021). However, it is currently not known if ECS can also be used to target subcortical regions.

Recently a groundbreaking study introduced a novel electrical brain stimulation technique that can target deep brain regions non-invasively using interfering fields (Grossman et al., 2017). This technique, interferential current stimulation (IF), utilizes the concept of wave interference between two sinusoidal stimulation sources of high but slightly different frequencies (e.g., 5,000 and 5,010 Hz) applied to two pairs of TES electrodes. The high frequency fields sum to generate, at a target subcortical region, a low frequency field equivalent to the difference between the two applied high frequencies (i.e., 10 Hz). The study used both computational and animal experiments to support the feasibility of this approach.

In a recent computational study on IF, Huang et al. (2020) used an array of TES electrodes for each stimulation source instead of only a pair of electrodes. This provides more flexibility to steer the electric field in the brain. In addition, they developed an algorithm to optimize the electrodes configuration to target a pre-defined subcortical region while avoiding others. Their results show a trade-off between electric field strength and focality. In addition, they demonstrated that the resultant electric field strength at any point had a maximal value equal to the lower electric field strength between the two sources at that specific point. In other words, the resultant low frequency field strength at the pre-defined target was limited to the strength of each of the two high frequency fields independently. Thus, an approach to increase the electric field strength of both sources in the brain would be valuable.

In this study, we hypothesized that IF using ECS electrodes would produce a stronger and more focused subcortical stimulation than IF using TES electrodes. To test this hypothesis, we used a human head computational model with either a set of 19 ECS electrodes or 19 TES electrodes. Then, we pre-defined a number of subcortical targets and calculated the optimized electrode configurations for each of the two approaches. Finally, we compared the fields’ strength and focality obtained with IF-ECS and IF-TES at the target points and showed that IF-ECS can target subcortical regions with more focused and stronger fields than is achievable with IF-TES.

## Materials and Methods

### Head Computational Model and Electric Field Calculation for Individual Electrodes

A high-precision human head computational model was generated using the multimodal imaging–based detailed anatomical (MIDA) model (Iacono et al., 2015) and then imported into ScanIP (Simpleware Ltd., Exeter, United Kingdom). The model consists of 115 head tissues and brain structures. These tissues may differ in their electrical conductivity values. However, only a limited number of them has been investigated and validated. Thus, it becomes necessary to simplify the model to a limited number of tissues with known and validated conductivity values. In this study, we followed a similar approach to the majority of the similar modeling studies by combining the different sub-tissues to form the five most essential and commonly used tissues: skin, skull, cerebrospinal fluid (CSF), gray matter (GM), and white matter (WM). This approach was validated by comparing modeling results to intracranial brain recording in humans and non-human primates and was shown to have similar results (Opitz et al., 2016; Huang et al., 2017; Lafon et al., 2017). Arrays of 19 TES or ECS disk (15 mm diameter) electrodes were added based on the 10–20 system. The location of the electrodes were calculated using the “Mesh2EEG” Matlab toolbox (Giacometti et al., 2014). The ECS electrodes were modeled with an insulating back layer that covers them and prevents direct contact with the skin (Khatoun et al., 2019). The electrode contacts were modeled as platinum disks and the insulating back layers as a polydimethylsiloxane (PDMS) silicon material, commonly used for invasive electrodes (Meacham et al., 2008; Guo et al., 2013; Ochoa et al., 2013). The insulating back layer had a 20 mm diameter and 1.6 mm thickness.

After tetrahedral meshes were generated using ScanIP, the volumetric model was imported to COMSOL multiphysics 5.3 (COMSOL, Inc., Burlington, MA, United States) to calculate the electric field distribution for each electrode separately. Thus, a total of 38 electric field simulations were required. Each simulation estimated the electric field distribution through one of the ECS or TES electrodes with a 1 mA (zero to peak amplitude) current being set as a boundary condition for the finite element problem. Electric current was applied between the selected electrode and a reference electrode defined as the bottom of the head representing a far body reference electrode (Khatoun et al., 2019). Finally, assuming a quasi-static approximation of Maxwell’s equations (valid for frequencies <1 MHz), Laplace’s equation was solved to estimate the electric field vector (E) at each element in the model:


(1) ∇⋅σ⁢∇⁡φ=0

(2) E=∇⁡φ

where φ represents the electrical potential vector E and the electric field vector. The electrical conductivities (σ) for the tissues were always: σ_Skin_ = 0.465 S/m, σ_Skull_ = 0.01 S/m, σ_CSF_ = 1.65 S/m, σ_GM_ = 0.27 S/m, σ_WM_ = 0.126 S/m, σ_PDMS_ = 10^–19^ S/m, σ_pt_ = 9.43 × 10^6^ S/m (Mark, 1999; Peters et al., 2001; Akhtari et al., 2002; Datta et al., 2009; Gabriel et al., 2009; Khatoun et al., 2019). The results for each of the 38 simulations were exported to Matlab (MathWorks, MA, United States) where the optimization method was applied.

### Resultant Electric Field During Multi-Electrode Stimulation

Above, the electric field vector was calculated for each electrode separately assuming that the current is only passed between the selected electrode and the reference. Here, the resultant electric field for simultaneous multiple electrodes stimulation is calculated. Using Matlab, the electric field distributions for ECS and TES electrodes were sorted in separate matrices A_N × m_ where m represents the number of electrodes and N the number of brain elements (n) multiplied by 3 (*N* = *n* × 3) taking into account the electric field at the different coordinate axes (i.e., x, y, and z). Given the matrix A we can now estimate the total absolute electric field (E) generated in the brain by the electrode arrays as following:


(3) E=A.s

(4) $$\mathrm{Such}\,\mathrm{that}\sum_{i=1}^{m}s_{i}=0$$

(5) $$\text{And}\sum_{i=1}^{m}\left|s_{i}\right|\leq {\text{I}}_{\text{max}}$$

where s is a vector of length m representing the applied electric current to each electrode. Eq. 4 ensures all currents entering the brain must exit and Eq. 5 ensures that the maximum applied electric current is below a maximum value I_max_. Therefore, by adjusting the values in vector s, the induced electric field in the brain can be steered. This is essential when aiming to stimulate with a maximum field a given brain area while avoiding others. To maximize the electric field at a target brain element, a similar approach to Huang et al. (2020) was used:


(6) $$\begin{array}{c} a\,r\,g\,\text{max}\,e\,A\,s\\ s \end{array}$$

where e is the desired brain electric field vector having length N and specifying which brain element to target and what the desired field direction (i.e., x, y, and z) is. Each element in the vector e has a value between zero and one with higher values indicating the target area. In this study, the target point was set to have a value of one and then to decrease in a Gaussian distance relation when moving away from that target. To minimize the electric field at non-target brain elements the following constraint on the non-target electric field power was applied to the optimization approach (6):


(7) $${||\Gamma \,A\,s||}^{2}\leq {\text{P}}_{\text{max}}$$

such that (| | .| |) is the L2-norm and Γ is a diagonal matrix with size N and having diagonal component with values between zero and one to specify the weight of each element in the penalty form. In contrast to e, Γ has a value of zero at the target and increases in a Gaussian distance relation when moving away from that target. P_max_ is an input parameter that specifies the maximum allowed electric field power in the non-target brain elements. A low P_max_ value restricts the electric field in the non-target region but might also limit the applied electric current leading to lower fields in the target. The default value of P_max_ was calculated as P_max0_ = eT A(A^T^ Γ^2^A)^–1^ A^T^e (Fernández-Corazza et al., 2020; Huang et al., 2020).

### Interferential Stimulation and Electrodes Configuration Optimization

So far it was assumed that the electric current applied to all electrodes was sinusoidal and from the same source, meaning that they have the same frequency and phase. However, when two groups of electrodes receive electric current from two different sinusoidal sources with slightly different high frequencies, interferential phenomenon occurs. This section aims to calculate the optimal electrode configuration, of both IF-ECS and IF-TES, to target any pre-selected point in the brain with the resultant interferential low frequency field. Thus, we sought to use an approach similar to Huang et al. (2020). Assuming s1 and s2 are the electric current vectors of the first and the second sinusoidal current sources, respectively, the main objective becomes to optimize them. At any given brain element, the resultant interferential electric field is equal to 2 × min (| | E_1_| |,| | E_2_| |) where E_1_ and E_2_ are the electric fields generated by the sources s_1_ and s_2_ at the given brain element, respectively. To maximize the resultant interferential field at the target, s1 and s2 were calculated by solving the following optimization problem:


(8) a⁢r⁢g⁢max⁢ 2⁢min⁡(||eT⁢A⁢s⁢1||,||eT⁢A⁢s⁢2||)s⁢1,s⁢2

(9) $$\mathrm{Such}\,\mathrm{that}\sum_{i=1}^{m}s_{1i}=\sum_{i=1}^{m}s_{2i}=0$$

$$\text{And}\sum_{i=1}^{m}\left|s_{1i}\right|\leq {\text{I}}_{\text{max}},$$

(10) $$\sum_{i=1}^{m}\left|s_{2i}\right|\leq {\text{I}}_{\text{max}}$$

To minimize the resultant interferential field at the non-target elements the following constraint to the above optimization was applied:


(11) $${||2\times \text{min}\,\left(||{\text{e}}^{T}\,{\text{As}}_{1}||,||{\text{e}}^{\text{T}}\,{\text{As}}_{2}||\right)||}^{2}\leq P_{\max}$$

### Targets Preselection and Comparison Parameters

To determine if IF-ECS can target subcortical regions and compare the focality and field strength of IF-ECS to that of IF-TES, a number of subcortical targets were preselected. The selected regions are the left subthalamic nucleus (STN), right ventral intermediate nucleus (VIM), left hypothalamus and the left insula, which are of clinical and research interest for neuromodulation. Each region was defined by one point. Then the optimal electrode configurations were calculated for both IF-ECS and IF-TES for a series of P_max_ values as described by Huang et al. (2020). Finally, the electric field strength and focality at the target points were compared between IF-ECS and IF-TES. To compare the focality, the half-value volume (F) was calculated (Deng et al., 2013; Khatoun et al., 2018, 2019). This is equivalent to the cubic root of the brain volume having an electric field magnitude higher than half of the electric field strength at the target.

### Limiting the Number of Epicranial Electrodes

Because IF-ECS works using minimally invasive electrodes, limiting the number of implanted electrodes becomes important. However, this comes at the cost of electric field strength and focality. To test the effect of limiting the electrodes number on such parameters, the values were recalculated with only the electrodes with the highest applied current accounting for at least 75% of the total applied current. Then the applied currents were proportionally readjusted to ensure a total of 1 mA current was injected. The selection was done for each of the two sources (s1 and s2) and for the cathode and anode poles separately.

## Results

For each of the pre-selected targets the optimal electrode configuration was calculated given a predefined desired electric field direction (*x*-axis). To adjust the balance between maximizing the interferential field at the target and minimizing the field at the non-target regions, the solution for Eqs. 9–12 was solved for several values of P_max_ varying between P_max0_ × 10^–3^ and P_max0_ × 10^5^ in a step of 10^1^.

### Subcortical Brain Stimulation Using Interferential Stimulation-Epicranial Cortical Stimulation

**FIGURE 1:**
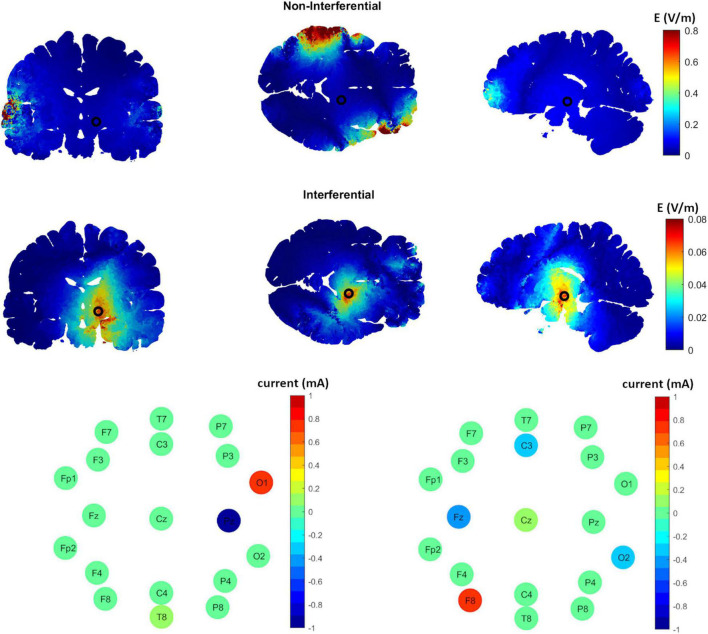
The distribution of the induced interferential and non-interferential electric fields and their corresponding electrodes configuration to target the subthalamic nucleus (STN) using epicranial electrodes. The first row shows the high frequency non-interferential electric field distribution along different brain sections (from left to right, coronal, axial, sagittal). As shown, the highest electric field values are on the cortex under the stimulation electrodes with much weaker fields at the center of the STN (indicated by black circle). On the other hand, the interferential fields (row two) show the strongest values at the target and weaker ones at non-target regions. The third row displays the corresponding optimal electric current distribution along the different electrodes for each of the two sources s1 **(left panel)** and s2 **(right panel)**.

Figure 1 shows an example of the results obtained using epicranial electrodes. The cross-sectional planes show a relatively high interferential electric field in the target region (STN, with the center indicated by a black circle) and a relatively low interferential electric field in the non-target region (second row, Figure 1). This is not the case for the non-interferential fields where the electric field is highest in the regions under the electrodes (first row, Figure 1); as was shown before (Vöröslakos et al., 2018; Alekseichuk et al., 2019). The electrode configuration for the corresponding results is shown in the third row of Figure 1. The pattern shows that most of the current is applied between electrodes O1 and Pz for the first source s1, and F8, Fz, C3, and O2 for the source s2.

### A Tradeoff Between Field Strength and Focality

**FIGURE 2:**
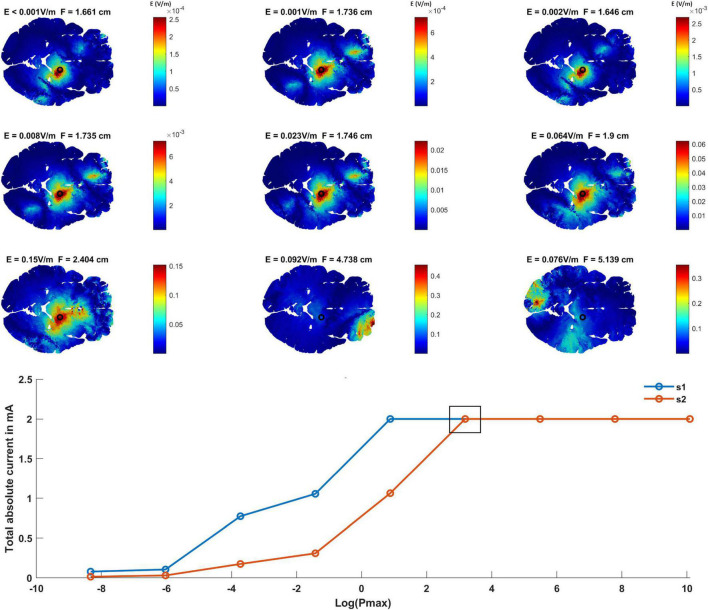
Finding the grand optimal interferential stimulation-epicranial cortical stimulation (IF-ECS) configuration for STN stimulation and studying the effect of increasing the maximum allowed electric field power (P_max_) on the induced electric field distribution. The first three rows show the interferential electric field distribution induced by the optimal electrode configuration as a function of increasing P_max_ (left to right and top to bottom). The electric field (E) value at the target (STN indicated by a black circle) and the focality (F) for each P_max_ value is indicated above each panel. As P_max_ increases, more current is allowed to be injected and more electric field is allowed at the non-target regions and the brain in general. However, this comes at the cost of focality with increasing P_max_ leading to a less focused stimulation (higher F values) at the target. The graph at the bottom of the figure shows the total absolute injected current for each of the two sources as a function of P_max_. As indicated, higher P_max_ values allow more current to be injected. The black box in the graph represents the grand optimal solution. This is defined as the solution for the lowest P_max_ value where the total absolute current of both sources has reached the maximum (1 mA zero to peak). This corresponds to the sixth panel (second row, third column) where *E* = 0.064 V/m and *F* = 1.9 cm.

The optimization method aims to maximize the interferential fields at the target and minimize it elsewhere. To adjust the leverage between the two factors, P_max_ was varied. Figure 2 shows the optimized interferential fields as a function of P_max_. Low P_max_ values show a more focused but weaker interferential field at the target. As P_max_ increases, the focality decreases and the strength of the field at the target increases. This is because as P_max_ increases, more electric fields are allowed in the non-target regions and thus more current can be injected. The graph at the bottom of Figure 2 shows the applied current amplitudes of each source s1 and s2 as a function of P_max_. The small square box indicates the grand optimal solution which represents the solution for the lowest P_max_ value where the total current applied to each of the two high frequency sources has reached maximum (i.e., 1 mA peak amplitude and 2 mA peak to peak). This solution was selected as the grand optimal configuration and was used for further analysis.

### Interferential Stimulation-Epicranial Cortical Stimulation vs. Interferential Stimulation-Transcranial Electric Stimulation

**FIGURE 3:**
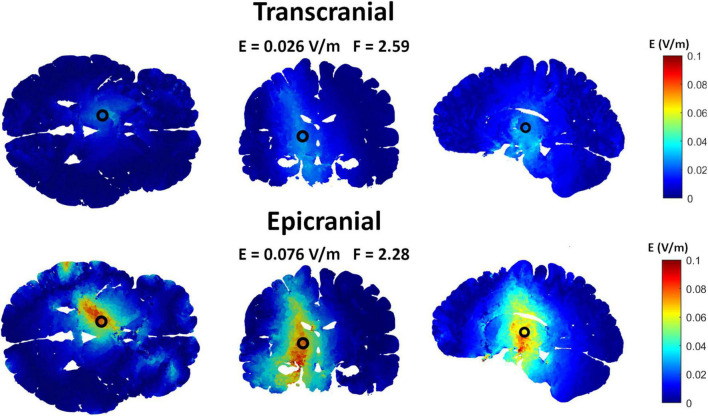
Comparing interferential stimulation (IF) using transcranial electric stimulation (TES) electrodes to that using epicranial cortical stimulation (ECS) electrodes to target the ventral intermediate nucleus (VIM). The upper row shows the electric field distribution obtained by the TES grand optimal electrode configuration while the lower row shows that obtained by the ECS grand optimal electrode configuration. Using ECS electrodes, the interferential fields show higher and more focused electric field compared to that using TES electrodes.

Epicranial cortical stimulation delivers stronger and more focused non-interferential electric field than TES (Khatoun et al., 2019). To test if this also applies to interferential fields, we compared the results obtained from IF-ECS to that of IF-TES. Figure 3 shows an example of the results obtained for each of the two techniques with the VIM being the target. The results show that IF-ECS produces more focused (2.28 cm vs. 2.59 cm) and stronger interferential fields (0.076 V/m vs. 0.026 V/m) than IF-TES, respectively. These results were similar for all selected targets (see Table 1). When the same stimulation amplitude (1 mA, baseline to peak) is used for the same target, IF-ECS shows an average of 3.8 times stronger and 9% more focused fields.

**TABLE 1:** Comparing the results of interferential stimulation (IF) using epicranial cortical stimulation (ECS) to that using transcranial electric stimulation (TES).

Target	Epicranial EField (V/m)	Transcranial EField (V/m)	Epicranial focality (cm)	Transcranial focality (cm)
STN	0.064	0.009	1.90	2.02
VIM	0.076	0.026	2.28	2.59
Hippocampus	0.037	0.019	2.10	2.35
Insula	0.064	0.008	1.86	1.94

### Limiting the Number of Epicranial Electrodes

**FIGURE 4:**
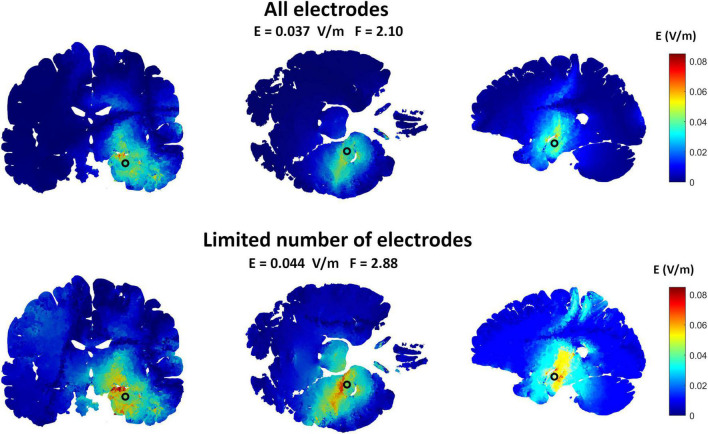
The effects of limiting the number of implanted ECS electrodes on the induced interferential field to target the hippocampus. The upper row shows the electric field distribution obtained by the ECS grand optimal electrode configuration when all the electrodes were used while the lower row shows the fields obtained when only the most essential electrodes were selected. The most essential electrodes were defined as the electrodes contributing to at least 75% of the injected current for both sources. This was a total of seven electrodes in this example. As shown, the fields are a bit less focused when the number of electrodes were decreased. But by readjusting the total amount of injected current to reach the maximum allowed values (1 mA peak amplitude), the electric field amplitude at the target was higher. This is in line with the amplitude-focality trade-off already discussed in this paper.

Figure 4 is an example comparing the results obtained using 19 electrodes to that using the major electrodes counting for at least 75% of the applied current (7 electrodes in this example). The results show a less focused and more spread electric field when using a smaller number of electrodes. On the other hand, the electric field strength is increased. Table 2 summarizes the results for all selected targets. The number of selected electrodes counting for at least 75% of the total current varied between 6 and 7 electrodes. On average, this resulted in a 28% less focused but 346% stronger fields.

**TABLE 2:** The effect of decreasing the number of electrodes on the electric field strength and focality.

Target	All electrodes electric field (V/m)	Limited electrodes electric field (V/m)	All electrodes focality (cm)	Limited electrodes focality (cm)	Number of electrodes
STN	0.064	0.10	1.90	2.68	6
VIM	0.076	0.08	2.28	3.18	7
Hippocampus	0.037	0.044	2.10	2.88	7
Insula	0.064	0.065	1.86	2.49	7

## Discussion and Conclusion

The first aim of this study was to use computational modeling to optimize the electrodes configuration and test the ability of IF-ECS to target subcortical regions. The optimization method we applied was already investigated in the context of TES and it was shown to successively work (Huang et al., 2020). In this study, the results from the applied optimization technique show that using IF-ECS it is also feasible to target subcortical brain regions while avoiding other brain areas. There was a tradeoff between the electric field focality and amplitudes. When more electric field was permitted in non-target regions, by setting higher P_max_ values, higher electric fields were reached in the target regions, but this comes at the cost of focality. This is in line with the findings using transcranial electrodes (Huang et al., 2020).

The second aim was to compare the outcomes of IF-ECS and IF-TES. It has already been shown that ECS induces a stronger and more focused cortical electric fields than TES. However, this is the first study to compare the interferential fields induced by both techniques. The results show that IF-ECS induces higher and more focused interferential fields than IF-TES. On average this was an increase of 3.8 times in field amplitude and an improve of 9% in focality. Moreover, ECS has the advantage that higher stimulation amplitude are achievable, due to the insulating back layer that blocks the current from passing through the skin (Khatoun et al., 2019). Therefore, a much stronger IF-ECS can be induced by increasing the stimulation amplitude above typical TES values. However, the advantages of IF-ECS come at the cost of invasiveness. IF-ECS would require a surgical intervention to have electrodes placed subcutaneously under the skin and directly over the skull. Thus, limiting the number of implanted electrodes becomes important. Our study shows that limiting the number of electrodes comes at the cost of electric field focality.

A limitation of this study is that it focuses on the induced interferential fields in the brain but not the non-inferential ones. However, this is based on our assumption that neurons will respond to low frequency electric fields but not to that with high frequencies. While there is evidence to support our assumption (Hutcheon and Yarom, 2000; Grossman et al., 2017), testing it is beyond the scope of this study.

Various studies have focused on the effects of anatomical differences and electrode dimensions on the electric field distribution during transcranial and epicranial stimulation (Laakso et al., 2015; Khatoun et al., 2019). A recent study investigated the effect of anatomical differences on the distribution of the interferential fields (Lee et al., 2020). Their results show that different subjects have different optimal montages to target the same brain region. Furthermore, the electric field values at the target varied between the subjects, but all subjects showed relatively stronger fields in the target compared to that in non-target regions. We believe that similar effects on the results would be obtained using epicranial electrodes. However, we did not investigate that in our study.

Epicranial cortical stimulation is a novel technique that was shown to induce strong and focused electric field in the cortex (Khatoun et al., 2019). This study shows that combining ECS with IF is a promising approach for delivering subcortical stimulation where no craniotomy is required. ECS is more invasive that TES but has the potential to deliver stronger and more focused electric fields in the brain. Thus, ECS and ECS combined with IF have the potential to fill a niche in the neuromodulation field for a number of clinical applications.

## Data Availability Statement

The raw data supporting the conclusions of this article will be made available by the authors, without undue reservation.

## Author Contributions

AK and ML: conceptualization, methodology, writing, and review. BA: methodology, writing, and review. All authors contributed to the article and approved the submitted version.

## Conflict of Interest

The authors declare that the research was conducted in the absence of any commercial or financial relationships that could be construed as a potential conflict of interest.

## Publisher’s Note

All claims expressed in this article are solely those of the authors and do not necessarily represent those of their affiliated organizations, or those of the publisher, the editors and the reviewers. Any product that may be evaluated in this article, or claim that may be made by its manufacturer, is not guaranteed or endorsed by the publisher.
